# Towards Health Equity and Transformative Action on tribal health (THETA) study to describe, explain and act on tribal health inequities in India: A health systems research study protocol

**DOI:** 10.12688/wellcomeopenres.15549.1

**Published:** 2019-12-13

**Authors:** Prashanth Nuggehalli Srinivas, Tanya Seshadri, Nandini Velho, Giridhara R Babu, C Madegowda, Yogish Channa Basappa, Nityasri Sankha Narasimhamurthi, Sumanth Mallikarjuna Majigi, Mysore Doreswamy Madhusudan, Bruno Marchal

**Affiliations:** 1 Institute of Public Health, 3009, II-A Main, 17th Cross, KR Road, Siddanna Layout, Banashankari Stage II, Bengaluru, Karnataka, 560070, India; 2 Vivekananda Girijaya Kalyana Kendra, Biligiriranga Hills, Yelandur Taluk, Chamarajanagar District, Karnataka, 571441, India; 3 Department of Ecology, Evolution and Environmental Biology, Columbia University, New York, 10027, USA; 4 Indian Institute of Public Health-Bengaluru, Public Health Foundation of India, Bengaluru, Karnataka, 560023, India; 5 Zilla Budakattu Girijana Abhivruddhi Sangha, Hosapodu, Biligiriranga Hills, Karnataka, 571441, India; 6 Ashoka Trust for Research in Ecology and the Environment, Royal Enclave, Sriramapura, Jakkur Post, Bengaluru, Karnataka, 560064, India; 7 Department of Community Medicine, Mysore Medical College and Research Institute, Mysore, Karnataka, 570001, India; 8 Independent Researcher, 3076/5, 4th Cross, Gokulam Park, Mysore, Karnataka, 570002, India; 9 Institute of Tropical Medicine, Antwerp, Nationalestraat 155, 2000, Belgium

**Keywords:** Health inequity, indigenous health, realist evaluation, participatory action research, tribal health, social determinants of health, forests and health

## Abstract

**Background: **In India, heterogenous tribal populations are grouped together under a common category, Scheduled Tribe, for affirmative action. Many tribal communities are closely associated with forests and difficult-to-reach areas and have worse-off health and nutrition indicators. However, poor population health outcomes cannot be explained by geography alone. Social determinants of health, especially various social disadvantages, compound the problem of access and utilisation of health services and undermine their health and nutritional status. The Towards Health Equity and Transformative Action on tribal health (THETA) study has three objectives: (1) describe and analyse extent and patterns of health inequalities, (2) generate theoretical explanations, and (3) pilot an intervention to validate the explanation.

**Methods: **For objective 1, we will conduct household surveys in seven forest areas covering 2722 households in five states across India, along a gradient of socio-geographic disadvantage. For objective 2, we will purposefully select case studies illustrating processes through which socio-geographic disadvantages act at the individual, household/neighbourhood, village or population level, paying careful attention to the interactions across various known axes of inequity. We will use a realist evaluation approach with context-mechanism-outcome configurations generated from the wider literature on tribal health and results of objective 1. For objective 3, we will partner with willing stakeholders to design and pilot an equity-enhancing intervention, drawing on the theoretical explanation generated and evaluate it to further refine our final explanatory theory.

**Discussion: **THETA project seeks to generate site-specific evidence to guide public health policy and programs to better contribute to equitable health in tribal populations. It fulfills the current gap in generating and testing explanatory social theories on the persistent and unfair accumulation of geographical and social disadvantage among tribal populations and finally examines if such approaches could help design equity-enhancing interventions to improve tribal health.

## Introduction

Over 8.6% of India’s population is comprised of tribal communities ^1^. Constitutionally recognized for affirmative action as Scheduled Tribes (STs), they are mostly forest-dwelling and have lived in and around legally protected forest areas in south, central and north-east India ^2^. Among various social categories in India, the ST population have poorer access to healthcare as well as poor national and state-level average population health outcomes ^3– 5^. These include higher maternal and infant mortality and morbidity due to communicable diseases, higher childhood malnutrition and higher rates of non-communicable diseases, which have increasingly been reported in recent years. Comparison of various demographic, health and nutrition indicators shows nearly uniformly poor health status among ST populations but with variations across Indian states (see *Extended data* for a table showing comparison) ^6, 7^. The social group Scheduled Tribe ** (ST) has its origins in state-specified lists for implementing affirmative action policies as per the Indian constitution. Several communities that may not have a close association with forests are also in this list. Hence, several tribal communities closely associated with forests prefer the term *Adivasi*. However, this is not true across the country and a common terminology covering India’s tribal communities is contested on linguistic, historical, ethnic and legal grounds ^8– 10^. Our hypotheses refer mostly to forest-associated tribal communities (hence *Adivasis*), but the term ST has been preferred for the purposes of this paper due to its widespread usage in health literature. At the stage of dissemination in reports and peer-reviewed journals, we may reconsider this terminology based on our interactions, to reflect how the communities' preferred labels.

Poor tribal health status in India mirrors a global pattern of worse-off health status among indigenous populations. A comprehensive meta-analysis of health outcomes in 104 million global tribal populations found that health, education and development indicators of Indian tribal populations are consistently poorer across the country, despite overall improvements in population health across Indian states ^6, 11^. This reflects a complex interplay between the socio-political, economic, and cultural conditions that contribute to this situation ^9, 11^. There is a disparity in health outcomes of tribal communities compared to non-tribal populations, as well as disparities within and across tribal communities ^6^. This is true even in otherwise better performing Indian states (in terms of health services performance and coverage) such as Tamil Nadu and Kerala ^6, 12^. Hence, research in tribal health needs to generate context-specific evidence that could be used to design and deliver locally targeted interventions to reduce inequities. Further, a better understanding of the heterogeneity of inequity patterns and of the processes driving these inequities must guide the framing of policies and programs related to tribal health at state and national levels. The aim of the project titled “Towards Health Equity and Transformative Action on tribal health” (THETA) is to generate context-specific local evidence to guide action, as well as generate wider theoretical explanations on drivers of inequities in tribal populations.

### Inequity and inequality

According to the World Health Organization, inequities are “unjust differences in health between persons of different social groups, (which) can be linked to forms of disadvantage such as poverty, discrimination and lack of access to services or goods” ^13, 14^. A related term, health inequality, needs to be distinguished from health *inequity*. Whereas inequalities in health are related to differences between population groups, arising from genetic, biological or other factors that may be randomly distributed, inequities, on the other hand, have a strong social causation and a non-random pattern of distribution; they tend to aggregate in specific socially constructed groups due to underlying societal characteristics that mediate access to power and resources ^14, 15^. The conceptual underpinnings of inequities is in social justice and in line with this, health inequities are characterised by: (a) systematic and consistent patterns of advantage or disadvantage across specific population groups (pattern of consistent differences in access to health services between rural and urban populations); (b) social, rather than biological, processes (nearly global pattern of higher mortality among low-income groups, a pattern observed across countries and over time); and (c) originates from and sustained by unjust social arrangements, resulting in an unequal distribution of the resources essential to achieve or maintain good health ^15, 16^. Health inequity is a normative concept that does not lend itself to measurement. Hence, health inequities are assessed by monitoring health inequalities; observable differences between subgroups within a population and identifying systematic patterns of these differences attributable to social phenomena ^13^. Since social processes underlie these health differences, we can expect that these gaps can be closed or significantly narrowed through suitable social policies and programs.

### Work leading up to this study

The THETA project builds upon two research projects implemented in three of the proposed study areas.

**Poverty traps study**: Velho *et al.* (2018) studied the relationship between forest dependence and socio-economic status of communities (including both ST and non-ST communities) living in and around forest areas (tiger reserves), namely Biligiriranga Swamy Temple (BRT), Kanha and Pakke, in Karnataka, Madhya Pradesh, and Arunachal Pradesh, respectively ^17^. These states differ in terms of overall governance, health systems performance and implementation of forest rights for tribal populations. We found that within the same geographical area and at a finer scale than is available through nation-wide representative surveys, tribal and non-tribal communities differ in terms of access to and utilisation of healthcare. Besides, we were able to characterise inter-site (state) differences and hypothesise possible explanations for these differences.

**Participation for local action (PLA) project**: In 2013, as a part of the PLA project, the Institute of Public Health, Bengaluru (IPH) set up a research field station in BRT. The purpose was to explore possibility of embedded community-based research using health policy and systems research approaches to strengthen health systems in tribal populations. The PLA project used a participatory action research (PAR) approach ^18^ to identify barriers and strengthen implementation of maternal health programs for the *Soliga* tribal community in the district. It was conducted by an interdisciplinary team consisting of researchers, implementers, and members from the tribal community-based organisation of the *Soliga* people. The PLA project identified the need for a *health navigator* to facilitate care for tribal patients referred to higher centres. The project was funded by the WHO Alliance for Health Policy and Systems Research under their Implementation Research Platform and was piloted in BRT in association with the *Zilla Budakattu Girijana Abhivruddhi Sangha* (ZBGAS, the district’s indigenous people’s welfare association run by members of the *Soliga* people). Through multiple iterations of inquiry, we found the critical role played by social networks and various social determinants in determining whether tribal patients received timely and appropriate care ^19^.

## Objectives

**Figure 1.**
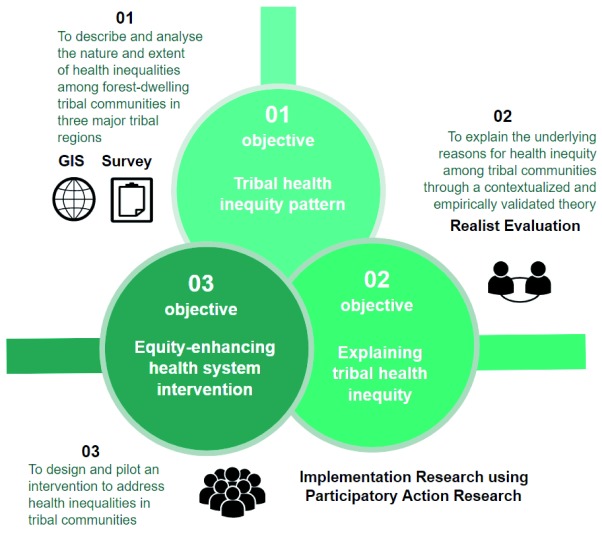
Objectives of THETA project in relation to methods.

We aim to document inequality patterns in major tribal regions of India and generate, validate, and test theoretical explanations for how social disadvantage could be driving health inequity. We will combine epidemiological methods with multiple health policy and systems research (HPSR) approaches in line with the three study objectives (see Figure 1). The field of HPSR is a question-driven functional research tradition that leans on multiple social science disciplines; researchers choose the method that is best suited for the purpose of achieving a socially relevant goal, which is set within a socially constructed health system setting, in the process acknowledging possibly diverse philosophical bases underlying the research methods ^20, 21^ According to Sheikh *et al.* (2011), “the range of questions encompassed by HPSR is broad....there are different levels of analysis—macro-level analysis analyzes the architecture and oversight of systems, meso-level analysis focuses on the functioning of organizations and systemic interventions, and micro-level analysis considers the roles of individuals involved in activities of health provision, utilization, and governance, and how systems respectively shape and are shaped by their decisions and behaviour” ^20^.

## Research questions

For each objective, the hypotheses we examine are given below. Detailed methods, tools, and analysis for each objective follow in subsequent sections.

### Objective 1 (patterns)

To describe and analyse the nature and extent of health inequalities among forest-dwelling tribal communities in three major tribal regions;

a) Tribal communities have poor health and nutrition status indicators when compared to non-tribal people in the same area

b) Remoteness alone does not explain this difference in health and nutrition status indicators

### Objective 2 (processes)

To explain the underlying reasons for health inequity among tribal communities through a contextualized and empirically validated theory. Here, a theory is meant as an explanatory abstraction at the middle-level between micro-level working hypotheses and broad overarching social science theories ^22^. Hypothesis building will be in the form of context-mechanism-outcome configurations that are derived from middle-range theories and working theories that we shall formulate based on empirically derived patterns and borrowing from wider social theories (see phase 2 under Methods).

### Objective 3 (action)

To design and pilot an intervention to address health inequity in tribal communities. The design of the intervention shall be based on a (program) theory that draws from the refined theories from studying the processes for inequities, and hence the intervention is also an opportunity to validate/refine the program theories on processes driving inequities. This step shall follow a participatory action research approach with the ZBGAS, implementers (health managers and health workers from the district) and local non-governmental organisations, allowing for shared agenda-setting and co-production of the intervention along with communities and implementers. This objective builds upon previous experience of researcher-implementer-community engagement in the PLA project ^19^.

## Methods

### Study design

For the three objectives, we shall use three distinct methodological approaches in line with best methodological approaches in relation to research question typology in health policy and systems research ^20^. We use epidemiological methods for the descriptive and explanatory questions (objective 1), realist inquiry for the explanatory question (objective 2) and participatory action research and implementation research methods for objective 3. Overall, the study design is a multi-method interactive study in three phases, each sequentially mapping onto the three objectives. In phase 1, we will conduct cross-sectional survey (patterns), followed by realist inquiry in phase 2 (process), and participatory action research with health services and community partners (action) in phase 3. The detailed methods of the three phases are below. Although phase 3 was envisioned originally to follow phase 2, we propose to begin both phases simultaneously in order to ensure sufficient time for both their outcomes to be developed and disseminated before the end of the THETA project. We foresee a mutual learning between the people and the activities involved in the *processes* (phase 2) and the *action* (phase 3).

The three different methodological approaches we use have distinct ontological and epistemological foundations and shall hence be described in three different phases corresponding to the three objectives:

1. Phase 1: Cross-sectional survey for various tribal health indicators (Objective 1)

2. Phase 2: Realist evaluation (Objective 2)

3. Phase 3: Participatory action research with health services and community-based organisation (CBO) partners. (Objective 3)

### Phase 1

**Figure 2.**
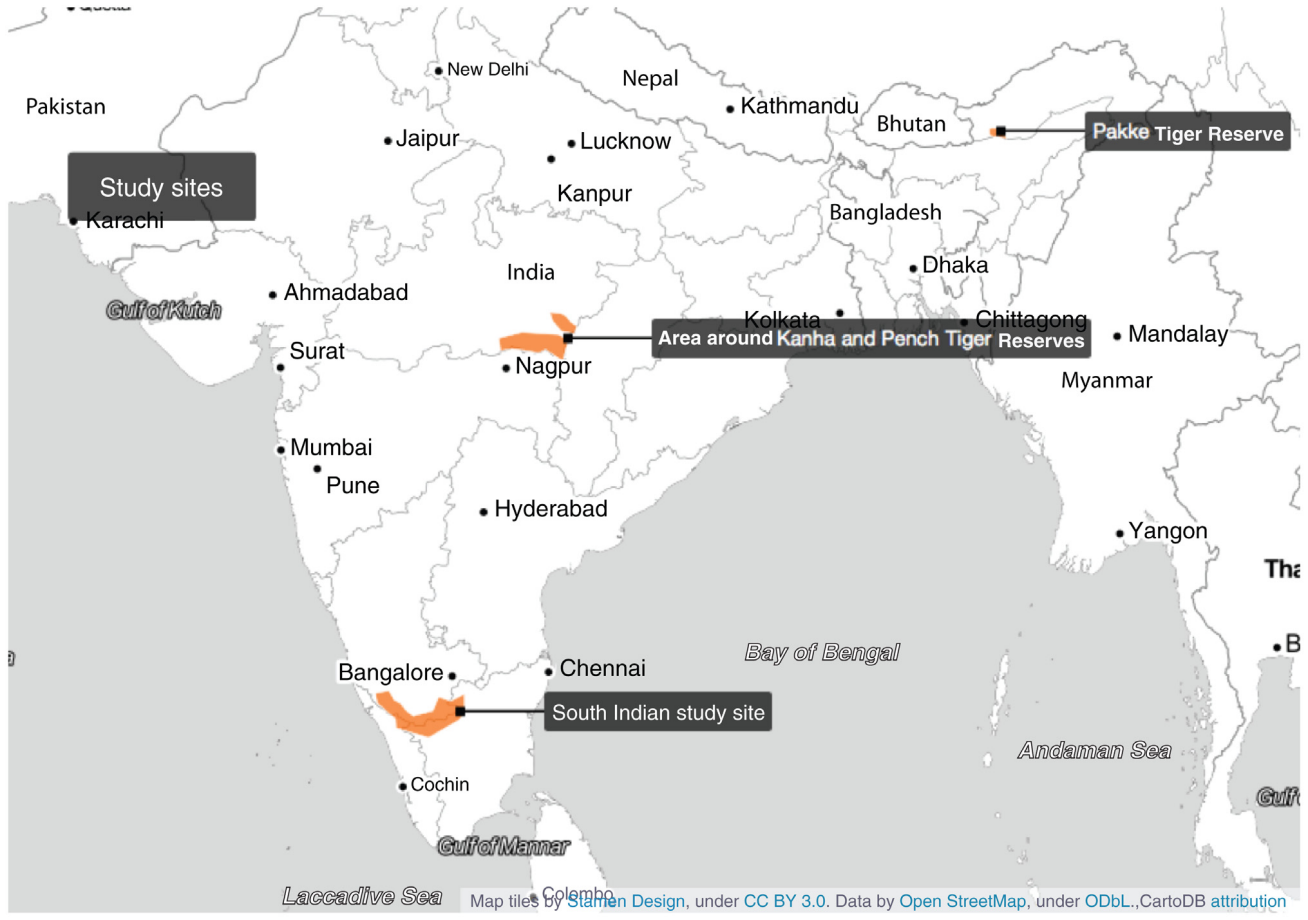
Field areas for the surveys in three different tribal regions spanning five states. Map tiles by Stamen Design, under CC BY 3.0, Data from OpenStreetMap. OpenStreetMap is open data, licensed under the Open Data Commons Open Database License (ODbL) by the OpenStreetMap Foundation (OSMF). Modified using CartoDB software.

**Summary of phase 1**: We will conduct a household survey of tribal and non-tribal households in three areas with tribal populations; Madhya Pradesh in central India (CI), Arunachal Pradesh in northeast India (NE) and Nilgiri forest area at the junction of three states in southern India ( Figure 2). We will select both tribal and non-tribal households in a representative manner using a geographical information system (GIS) based on a decreasing gradient of socio-geographical disadvantage index (SGDI), calculated using several village-level variables that combine social, environmental and geographic attributes. The survey questionnaire will include standardised and tested tools (see *Extended data*) ^23^ to assess maternal and infant deaths (mortality), illness profile (morbidity), and diet and anthropometry (nutritional status indicators). We will also collect data on individual and household level variables for socio-demographic characteristics, access and utilisation of healthcare, healthcare expenditure and health-seeking behaviour.

**Study setting:** We shall establish temporary field stations in central and northeast India, while in southern India, the field station at Biligiriranga hills (BR Hills) in Chamarajanagar district will oversee activities in the south Indian sites. In each of the five states, we will identify sites which correspond to protected area boundaries. We will choose seven sites: three sites in Karnataka, one site each in Tamil Nadu, Madhya Pradesh (Kanha Tiger Reserve), Kerala (Wayanad Tiger Reserve) and Arunachal Pradesh (Pakke Tiger Reserve).

The Chamarajanagar district of southern Karnataka lags behind most other districts in terms of development indicators. It also has a relatively large area classified as protected area under the Wildlife Protection Act 1972, including Bandipur and Biligiriranga Hills (BR Hills), both tiger reserves, and the Malai Mahadeshwara (MM Hills) wildlife sanctuary. Together, with contiguous forests areas in neighbouring states of Tamil Nadu and Kerala, these forests are part of the Nilgiri Biosphere Reserve with over 5000 sq. km of forests and at least 18 tribal communities. Table 1 is a list of tribal communities who will be included in the survey across all sites.

**Table 1.** Scheduled Tribe (ST) community sites to be covered under objective 1 of Towards Health Equity and Transformative Action on tribal health (THETA) project.

Sl. No.	State	Number of forest sites	Possible sites	ST communities covered
**1**	Karnataka	3	Malai Mahadeshwara wildlife sanctuary, Biligiriranga Swamy Temple tiger reserve & Nagarahole tiger reserve	Soliga, Jenu kuruba, Betta Kuruba
**2**	Kerala	1	Thirunelli Panchayat and surrounding forest area	Kattu nayakas, Paniyas, Adiya, Kuruchiya
**3**	Tamil Nadu	1	Erode/Nilgiris	Soliga, Irula, Badaga, Kota, Kurumba, Toda
**4**	Arunachal Pradesh	1	Pakke tiger reserve	Nyishi, Aka
**5**	Madhya Pradesh	1	Kanha tiger reserve	Gond, Baiga, Mariya

**Sample size:** A site is typically a single protected area (tiger reserve or wildlife sanctuary), which could span multiple administrative sub-divisions ** of a district or could be across few districts. For the purpose of sample size calculation, we considered prevalence of severe stunting among Adivasi children and anaemia among women in the 15–49 age group, as reported by the latest National Family Health Survey (NFHS) ^24, 25^. We considered ST status as being an attribute/explanator of poor health outcomes (an exposure). We used the standard sample size formula for cross-sectional/cohort study design on OpenEpi tool ^26^. We calculated a sample size that will allow us to make assertions about a given health outcome of interest (say the proportion of stunted children or anaemic women) among tribal and non-tribal populations within and across sites. The difference in the prevalence of severe stunting between Adivasi tribal and non-tribal rural children was estimated to be 9% (29% among Adivasi vs 20% among rural children). From NFHS-4 data for India, we calculated overall samples size covering all sites for anaemia (rural women 54.2% vs ST women 59.9%, giving a risk ratio of 1.1). Between these two, we adopted the sample size from anaemia as our study sample size as it is the higher one. The higher risk ratio for severe stunting in the UNICEF report is more likely to be a realistic estimate of the difference between Adivasi and rural households than the NFHS, because Adivasi is not a separately defined group in NFHS-4 (ST includes both forest-dwelling and other communities). Hence, the sample size estimation from NFHS-4 is likely to be a higher one as we expect a higher risk ratio between the tribal and non-tribal population in our survey. Assuming a 95% confidence interval, 80% power and an alpha error of 0.05, an overall sample size of 2474 individual women is estimated. We added a 10% non-response rate to this and obtained a total sample size of 2722. We shall attain this sample size across seven sites, giving an average of 388 per site. The final dataset will include other individuals interviewed at the households (in addition to the primary respondent) and will hence be larger than the desired sample size (including the men who were present at the time of survey and children).

**Figure 3.**
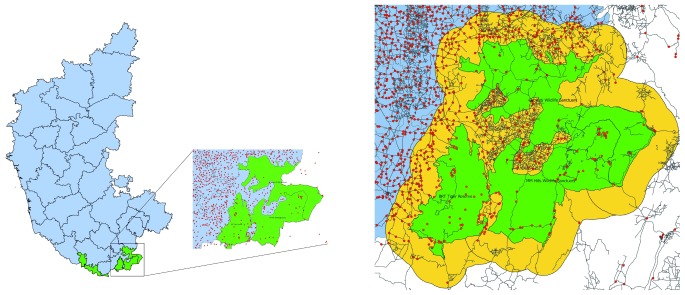
Left panel shows three sites in Karnataka; right panel is close-up of two southern Karnataka sites showing human settlements as red dots (both tribal and non-tribal) within green area (protected area of tiger reserves) and yellow area (buffer zone from the edge of the protected area that is included in our sampling); black lines show metaled roads.

**Sampling strategy:** A multi-stage sampling strategy shall be used. At the first stage of sampling, a list of the tribal and non-tribal villages will be selected from 2011 Census enumeration areas in each site and mapped in a geographical information system (GIS) platform using QGIS software ^27^. A vector layer of protected forest area boundary shall be imported into QGIS and an additional vector layer of buffer zones from the edge of the protected forest area will be created ( Figure 3). The buffer zone area shall vary from site to site depending on an assessment of forest dependency and perceived effects of nearby protected areas on livelihoods and other socio-economic characteristics. This shall be determined based on a discussion with local researchers and other stakeholders. We estimate a smaller buffer zone in southern Indian sites (between 5–10 km) and larger one in CI and NE sites (10–20 km). 

**Figure 4.**
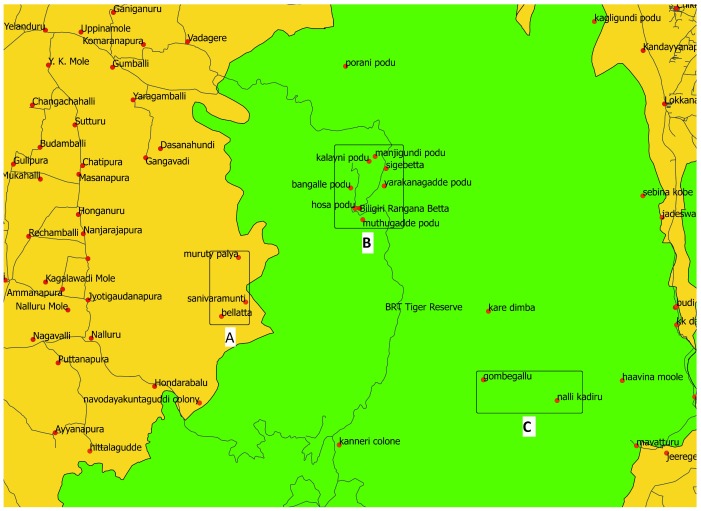
Choosing location of settlements based on a measure of socio-geographical disadvantage helps prevent us from treating settlements in Box B as if they are remote (well-connected despite being within tiger reserve). In this figure, settlements marked in Box A and those in Box C are much more disadvantaged than those in Box B.

**Participant selection and recruitment:** All villages and settlements within and outside the protected area boundary up to the designated buffer zone shall be selected. For all selected villages, we shall create an aggregate index score of socio-geographical disadvantage using a list of pre-identified variables. We will begin with correlated variables of geographical access. These include public transport travel time to nearest municipal administrative city, district administration, access to a high school/secondary school, primary health centre, tertiary hospital, walking travel time to all-weather motor road, sub-centre, population (government health facility below a primary health centre typically catering to a population of about 3000–5000 people, usually across few villages), altitude, rainfall, forest thickness, proportion of houses with a supply of improved drinking water, proportion of houses with an electricity supply. We will identify strata (or groups) of settlements that have shared socio-geographical advantage/disadvantage parameters based on principal component analysis, striving for intra-strata homogeneity while ensuring inter-strata/group heterogeneity with respect to disadvantage. Such homogenous strata/group shall be the primary sampling units. We foresee 3–4 such strata per site, covering groups of villages in remote or core forest areas (typically exclusively tribal), villages at the edge/outside forest, but also groups of villages that may be inside the protected area but very well connected by all-weather roads (typically mixed tribal and non-tribal population), and groups of relatively well connected villages in the plains (tend to be typically non-tribal) (see Figure 4).

For each site, three strata corresponding to low, medium and high percentiles of an index of socio-geographic disadvantage (SGDI), determined based on their clustering together with respect to the index scores will be identified and one-third of the sample size for that site shall be allocated to each stratum. Then, we will list the villages/settlements in that stratum and randomly choose one-third of these as our secondary sampling unit. The number of households to be sampled within each selected villages/settlements will be calculated in proportion to the population size in that village/settlement.

In the case of tribal villages, the local tribal community-based organisation (such as ZBGAS in southern Karnataka) will be approached for household details in each settlement, whereas for census/revenue villages, the local *gram panchayat* (the lowest level of local government at the village level in the decentralized government structure established by law in many Indian states) will be approached for these details. Then, for each village, a sampling interval shall be calculated. Depending on the number of households to be recruited in a given village/settlement, a random starting point from the centre of the village shall be defined and then every n-th household (n being the sampling interval) will be approached. Wherever the approached household is unavailable/does not consent to participate, the next available and consenting household shall be chosen.

For each selected household, a trained team of two data collectors shall conduct the survey. Upon approaching the household, the team shall invite any household member who is able and willing to provide information and obtain an audio-recorded verbal consent. After obtaining consent, respondents from the household shall be identified for the questionnaires listed in Table 2. Members who are ill or are unable to provide consent shall not be included in the study.

**Table 2.** List of tools and their corresponding respondents.

Sl. No	Module	Administered to
**1**	Socio-demographic characteristics	Head of household
**2**	Women’s health (1)	Ever married women in reproductive age group (15–49 years) who had a pregnancy/childbirth in the last year
**3**	Women’s health (2)	Ever married women in reproductive age group (15–49 years) who did not have pregnancy/childbirth in the last year
**4**	Child health and development	Mothers with children under five years of age
**5**	Anthropometry	All members of the household
**6**	Blood tests	All adult members of the household

**Figure 5.**
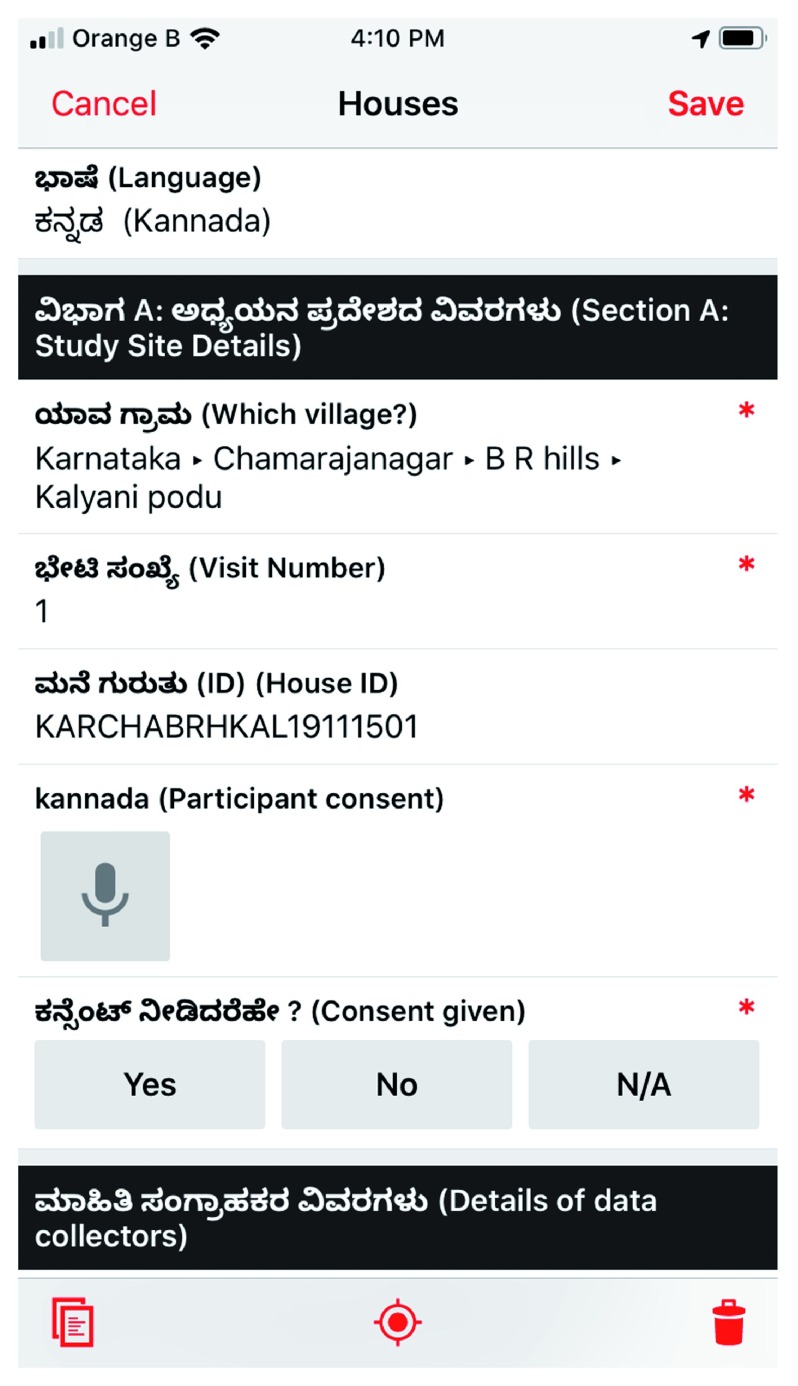
Screenshot of the app showing automatic unique ID generation and language localisation. Copyright 2019 Fulcrum Community / Spatial Networks Inc.

**Procedures for data collection:** We shall collect both primary and secondary data. Primary data shall include (1) survey responses using a questionnaire, (2) measurements for anthropometry and, (3) blood samples for clinical parameters. For phases 2 and 3, primary data shall include observational data, narratives captured during in-depth interviews, media files for case studies during the theory-driven inquiry, and intervention monitoring data under phase 3. Electronic tablets will be used for data collection. We will use a custom-made app using the Fulcrum cloud-based mobile data collection platform with offline data collection, multiple Indian language support and cloud-sync support to collect data during the survey. Alternately, Open Data Kit provides a suite of free and open-source tools that could be used to achieve comparable results. The survey questionnaire shall be administered using the mobile tablet-based app (see Figure 5).

Data collectors will be recruited locally (from within/nearby districts) and trained in the use of the tablet and the mobile application as well as the administration of the survey questionnaire during a five-day workshop that will be separately held in each data collection site. Two rounds of piloting will be conducted; the first one within the team and another in the field among non-sample households. Following the piloting, we anticipate that the questionnaires may need minor changes which will be incorporated.

Photographs of villages will be taken to document access, hygiene, living conditions, etc. without identifying information of houses/individuals. In the sampled households, data collectors will screen a video recording to the household members and then seek verbal consent. Verbal consent will be sought due to the low literacy rates in the study sites and our experience with people’s apparent comfort with discussing and clarifying consent orally rather than affixing their signatures onto previously printed text. Recorded verbal consent from the participant will be set as a prerequisite before administration of the questionnaires. The household questionnaire will begin with an initial module on socio-demographic household characteristics followed by other modules involving the following respondents: (a) youngest ever-married woman in reproductive age group (15–49), (b) her partner/husband (failing that, another adult male from the household between 15–60), (c) mothers of children below five and (d) all children for anthropometry. The questionnaire will have in-built skips, jumps and validity checks. At the end of the survey, the data collectors will seek consent for anthropometry tests from all members of the household who fulfil the inclusion criteria (assent from children in addition to mother/guardian consent). Permission for anthropometry shall be asked from children above 12 years of age. Anthropometry includes measuring the height (length for infants <1 year old), weight (measured using a standardised digital weighing scale), head circumference and mid-upper-arm circumference (for children <5 years), waist and hip circumference (using standardised measuring tape). Height will be measured using a stiff measuring tape.

**Biological data:** In two of the southern Karnataka sites, a trained health worker shall visit the households where the survey was conducted and invite an adult respondent of the household survey (identified randomly in advance using the Kish method ^28^) to participate in biological data collection. The reasons, procedure and benefits of blood tests will be explained to them and their consent re-established via a verbal consent process similar to the survey data obtained above. That the results of the tests will be made available to them will be explained clearly. Based on the information provided, they can choose to participate or refuse to do so in this component of the study. The health worker will measure the blood pressure of the participant, and ensure optimal general health status, enquire about any long-term medicine use and chronic disease. After explaining the procedure of drawing blood, under aseptic precautions, they will collect 5ml of blood in fasting state from superficial veins in the elbow of the participant. Of the total 5ml collected, 3ml of blood will be collected in plain vacutainer tubes and 2ml in ethylene diamine tetra-acetic acid (EDTA) tubes. The whole blood sample will be used to test for haemoglobin using a handheld point-of-care testing device (Hemocue) and for fasting blood glucose (FBS) using handheld glucometer (Accuchek). If FBS is >110mg, we will provide 75g sugar and test after two hours for post-prandial blood sugar (PPBS). For PPBS, we will obtain 2ml of blood and prepare 1 aliquot of serum after centrifugation. The team shall set up temporary work stations for field-level processing of samples prior to approaching settlements for data collection. Such sites need to have a safe space for processing as well as stable electric supply for the centrifuge. The results of Hb, FBS and PPBS will be delivered to the household.

The health worker will ensure clotting at the site of venipuncture before moving to the next household. All samples will be labelled with unique identification numbers at the site itself and transferred to vaccine boxes with ice packs for transport. Within one hour, the samples shall be centrifuged for separation of serum. Both EDTA samples and the serum shall be stored in vaccine carriers with ice packs and the serum sample will be transported to a deep freezer. An interim storage site (typically a primary health centre or government hospital) that has 24-hour deep freezer facility and is accessible within half hour (by vehicle) of the processing shall be identified in collaboration with the district health and family welfare department and government health services. Pooled samples will be sent to the laboratory for testing (transport time not exceeding one hour). A few (2–4) aliquots of plasma and serum shall be stored in a bio-repository for future analysis in 500μl cryovials. The reason for storage of biological material is to minimise potential discomfort and optimise research costs involved, in case there is a need to obtain biological data again for testing of other hypotheses in the future.

**Tests to be performed**

**From serum**: Lipid profile (total cholesterol, triglycerides, very low density lipoprotein, low density lipoprotein and high density lipoprotein cholesterol levels), FBS and PPBS will be analysed using a fully automated analyser using spectrophotometric principles.

**From plasma**: Genetic analysis will be conducted to assess mutation for sickle cell disease.

In all sites outside Karnataka, we will use non-invasive point-of-care testing devices for haemoglobin estimation.

**Data collection tools:** The data collection tool kit consists of three modules (see *Extended data* for the tools) ^23^. The modules are adapted from widely used standardised household and woman’s survey questionnaires used in district level household survey and the NFHS ^25, 29^, Integrated Disease Surveillance Program (IDSP) non-communicable diseases risk factor survey questionnaire ^30^ based on the WHO-STEPS tool ^31^.

### Phase 2

**Figure 6.**
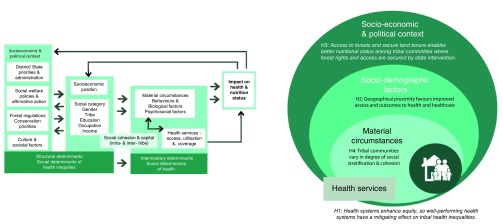
On the left panel, the framework proposed by the WHO Commission on Social Determinants of Health adapted for our study (especially at the level of socioeconomic and political context and at the interface of structural and intermediary determinants; illustration of possible hypotheses that we will test in objectives 1 & 2 drawing from the SDH framework. Initial phase of objective 2 will add more hypotheses based on literature (initial MRT).

The second and third phase will be conducted in Karnataka and Kerala sites with a more limited engagement in Arunachal Pradesh in northeast India. The focus will be on using realist inquiry ^32^ to build a plausible theory that explains tribal health inequality patterns. We shall begin with a set of hypotheses, drawing from the WHO’s social determinants of health framework, which includes various drivers of inequities in health, adapted to the south Indian regional context (see Figure 6). We will then identify theories from wider social science literature to explain overall tribal development in India and create a conceptual framework that integrates contextual information from study sites as well as theoretical insights ^33^. We will then develop an initial middle range theory (MRT) from which sub-theories (program theories) and hypothetical frames in the form of context-mechanism-outcome statements (CMO configurations) can be formulated. We will purposefully select case studies that will use both quantitative data and qualitative data to develop, iteratively test and refine an explanatory theory in three to four cycles ^33– 36^. The preparation of initial MRTs as well as the CMO configurations will closely align with discussions within the research team across the three proposed sites, such that they will aid the design and implementation of the case studies.

**Figure 7.**
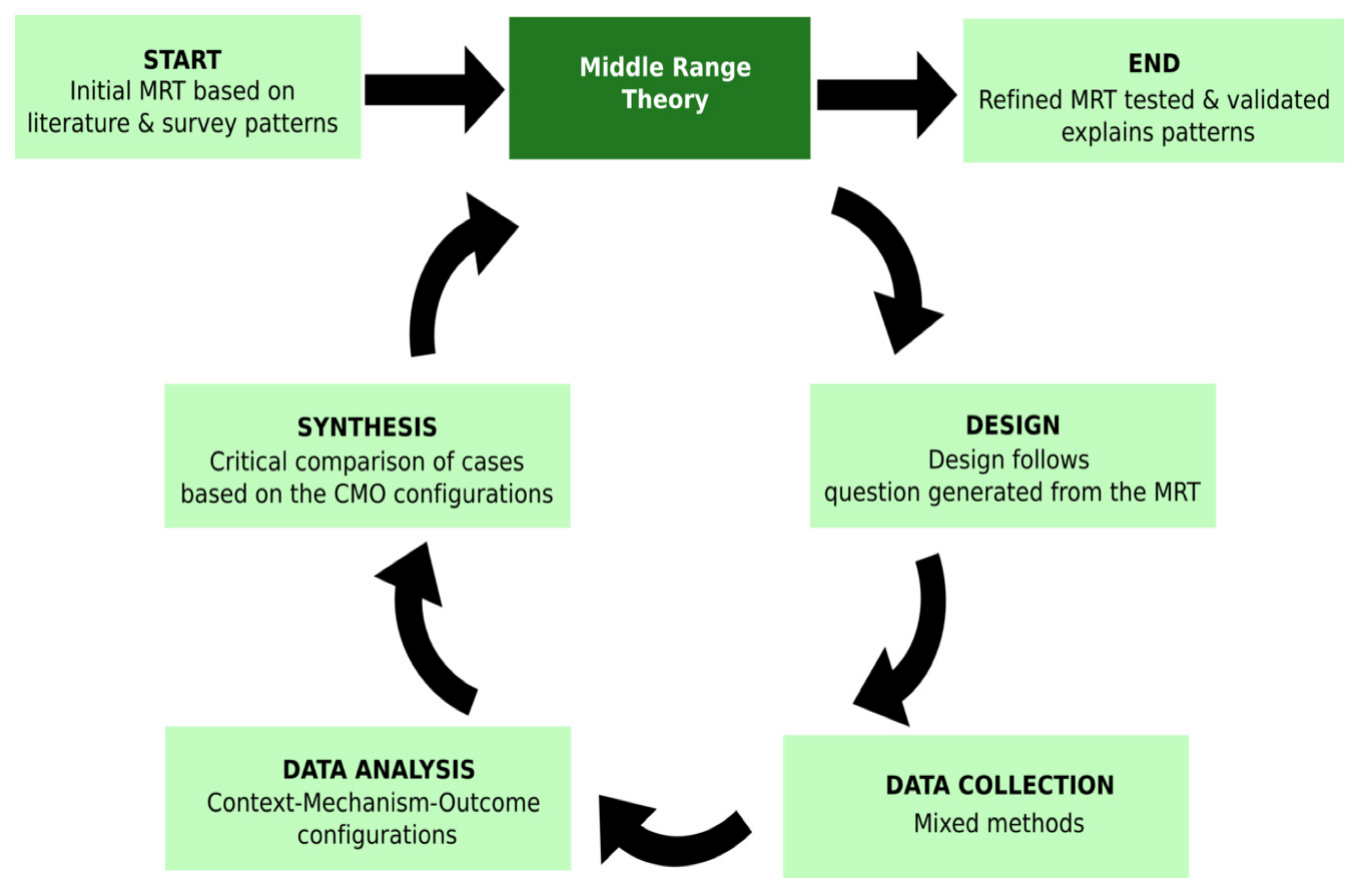
Illustration of the iterative nature of inquiry in realist approach.

The case studies shall focus on testing/refining the initial MRTs through three to four iterations ( Figure 7). At this stage, based on ongoing literature synthesis and preliminary results from three of the phase 1 cross-sectional surveys, initial MRTs are likely to focus on mechanisms of inequity across multiple levels ranging from governance (macro), health services (meso), community processes (micro) and their interfaces.

1. Explaining the contribution of historical and social factors in determining current geographical remoteness of a village
2. Explaining poor healthcare experience in secondary/tertiary care for tribal communities
3. Explaining intra- and inter-tribal differences based on site-specific inequality patterns observed in the survey
4. Contrasting above MRTs and their results in an area with a tribal majority (Arunachal) to explain/test if remoteness affects tribal communities similarly there as well.

**Site-based case studies:** We will develop case studies that focus on one or more of the following: geography (village/settlement or entire site), phenomena/experience (healthcare seeking experience in secondary/tertiary centre), socio-political role, ethnicity (Adivasi group) or at the interfaces between the community and non-governmental organisations (NGOs) etc. in order to further refine/test the initial MRT. Qualitative data using in-depth narrative inquiry, field notes and observational data will be used to prepare case studies.

**Refining the MRTs:** While each case study will aim to deepen the testing of one or more CMOs, upon completion of each site-based case study, we will refine, strengthen or refute (elements of) the initial MRT.

For phase 2, the sampling strategy shall be purposive. Each case study shall try to achieve a diversity of participants in terms of age, sex, location and social roles played in that society. Sample sizes shall be typically 4–10 participants (for in-depth interviews) in each case study, depending on the nature of CMOs designed in the initial MRT. The number in each case study could vary depending on the refining process. After each round of data collection and preliminary analysis, the next round of participants shall be determined based on the type of inquiry to be initiated. Data collection for each case study will be considered complete either on achieving saturation in terms of themes/content or upon achieving sufficient refining of the CMO. We will use the critical comparison of cases to also test hypotheses drawn from the social determinants of health (SDH) framework earlier at various levels (a few are illustrated in right panel in Figure 6).

### Phase 3

The third phase shall only be implemented in the Karnataka site. This phase will closely follow participatory approaches within the implementation research framework ^37^. We shall conduct multiple rounds of consultations with the ZBGAS, local NGOs and implementers based on the results of the cross-sectional survey and realist evaluation. In this process, we shall identify willing partners (either NGOs and/or a district administration or state partner interested in enhancing equity of their tribal populations) for co-production of one or few interventions broadly aimed at enhancing health equity (equity-enhancing health system intervention; EeHSI). An initial intervention design shall be offered for discussion among partners and will be adapted based on discussions with partners over a series of meetings/workshops. The MRT will allow the identification of entry points into addressing health inequities of the district’s tribal population. Some of the plausible entry-points are foreseen at three levels: health systems governance (agenda/priority setting at government/policy/institutional levels), health services (improving the interface between tribal communities and government health services), community (strengthening community-based platforms/structures to facilitate care or improve accountability of existing services). Current discussions with the ZBGAS based on initial exploratory workshops indicate the following possible directions for co-produced interventions.

A) Interventions focusing on health systems governance: Inter-sectoral action for health with *gram panchayats* covering tribal populations on implementing existing programs related to tobacco/alcohol use.
B) Interventions focusing on improving access and care with health workers and health services: Depending on insights from phase two, this could focus on interdisciplinary researcher-implementer-community platform for designing interventions for health problems specific to tribal populations and for health problems that are not yet being effectively responded to among tribal populations (cardiovascular disease care including stroke, chronic obstructive airway diseases, haemoglobinopathy, mental health including deaddiction for tobacco and alcohol). Interventions that strengthen care for non-communicable diseases at primary health centres catering to tribal populations or those that improve acceptability of care through improved cultural competence of health workers and hospital staff at distant/higher level hospitals where tribal patients are referred.
C) Community health and accountability: A package of interventions in partnership with the village health and sanitation committee and the ZBGAS to improve navigation across the health services and facilitation of benefits from various schemes designed for people below poverty line (such as the recently launched *Ayushman Bharat* scheme that seeks to provide free care at the point of service delivery but may not be easily accessible for marginalised populations such as tribal communities). Intervention could also focus on strengthening the leadership of the ZBGAS in engaging more effectively with the district and state governments to address specific health needs of tribal community.

**Figure 8.**
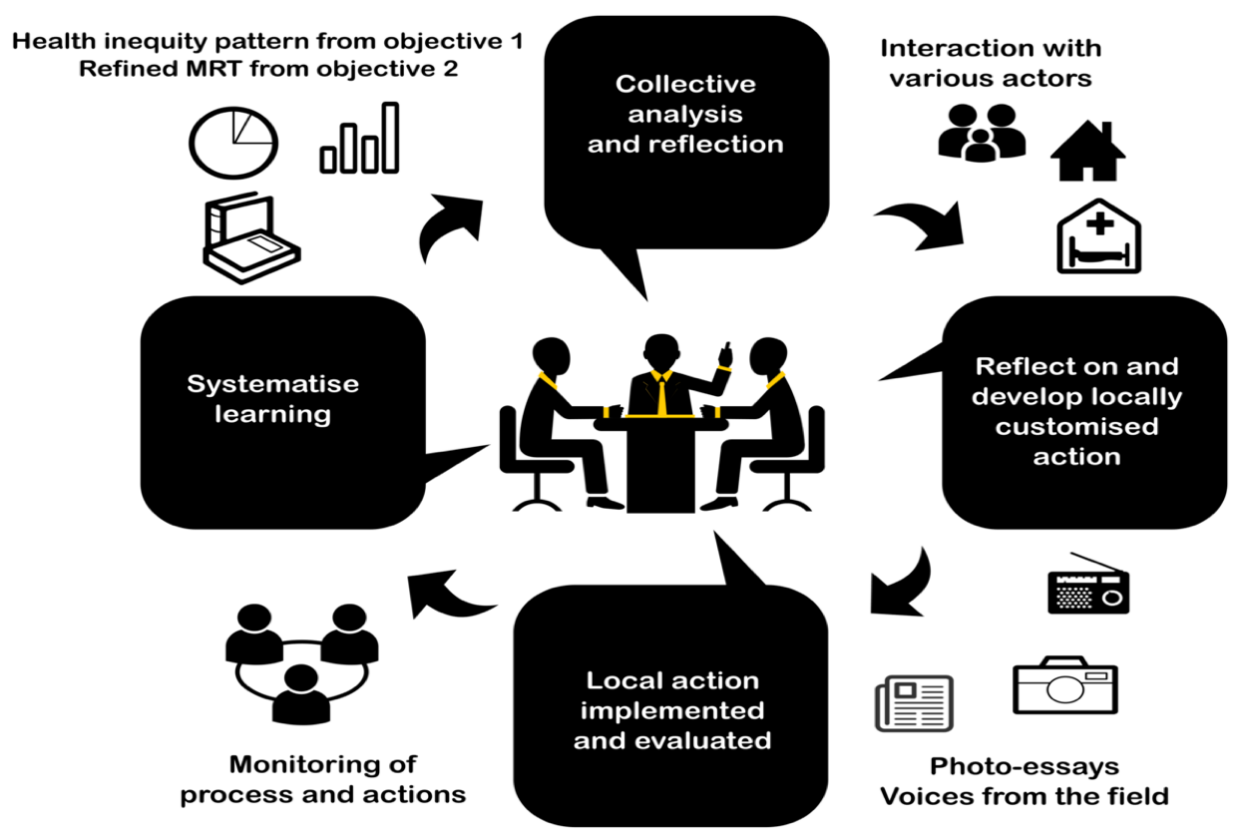
Action-reflection cycles in phase 3.

The design and implementation of EeHSI through action-reflection cycles that are characteristic of participatory action research cycles (see Figure 8), will also serve the purpose of validating the MRT. The qualitative data collection that began for objective 2 will continue during this phase. This phase will end with formulating how the intervention worked, for whom and under what conditions ^38, 39^. Data collected in phase 3 shall be (a) notes of consultative meetings and workshops, (b) intervention implementation data, (c) in-depth interviews of people involved in the intervention and field notes from observations. Anonymised secondary data related to the intervention implementation will also be collected.

We hope that the health inequity patterns and other data revealed by our study may help characterise the population and establish a long-term cohort. We are unaware of well-designed long-term cohort studies among tribal populations and this will go a long way in understanding causality of poor outcomes in tribal populations over time. Along with this, we aim to initiate an embedded participatory research agenda involving community-based organisations, implementers and researchers in a collaborative platform to design and implement context-specific interventions to mitigate health inequities.

## Analysis plan

### Phase 1

The analysis of the data shall be organized across multiple levels (individual, household, village, site/landscape).

At the village and landscape level, an index of socio-geographic remoteness for each village will be used to identify villages with similar socio-geographic characteristics using principal component analysis. Based on scores obtained from the principal component analysis, we will identify one or more indices that summarise different configurations of the input variables along multiple axes. This will allow comparison of villages in and around forests across and within sites (along these indices), as well as examine if and how village averages of health and nutrition outcomes vary along a gradient of socio-geographic remoteness. A geospatial analysis to examine correlates of geographical disadvantage in terms of poor health and nutrition outcomes, type of village (pre-dominantly tribal versus non-tribal) will be prepared for each site and patterns examined across sites to generate site-specific hypotheses to explain these patterns.

For the individual health parameters statistical analysis, we will track the following response variables: mortality rates (maternal and infant), nutritional status (body mass index, wasting and stunting), haemoglobin percentage, disease prevalence (hypertension). Intermediate outcomes such as access and utilisation of health services (outpatient care, inpatient care, maternal and child health), coverage rates (immunisation, select disease control programme indicators) and out-of-pocket expenditure will also be considered as response variables. We will model these response variables as a function of predictors such as protection regime (whether within protected area such as reserve forest/tiger reserve etc under the wildlife protection laws), generations/years since resettlement, distance to nearest road, nearest primary health centre, nearest town, proportion of forest cover. We will use Generalised Linear Mixed Models (GLMMs) to analyse data and apply an appropriate model-building approach to select a set of models that best explain health access and outcomes across tribal and non-tribal populations in relation to geographical access and/or social disadvantage.

### Phase 2

The analysis approach has been explained earlier. Through iterative insights built from the case studies and the refining of the MRT, we will generate a refined MRT that explains the site-specific inequity patterns with analytical generalizability to similar tribal population contexts. The case study series coupled with quantitative data analysis from the cross-sectional survey will provide us with a systematically developed body of knowledge of the underlying causes of relative social disadvantage within and across tribal communities, as well as with nearby non-tribal communities.

### Phase 3

Detailed documentation of the agenda-setting stage and the future iterations of participatory inquiry with the community-based organisation and other stakeholders shall be conducted. The intervention shall be monitored and a qualitative inquiry conducted to examine if, and how, the intervention addressed one or more drivers of inequities in this population. Multiple iterations of action-reflection cycle shall be attempted in line with the PAR approach.

## Ethics

Phase 1 has received ethics approval from the institutional ethics committee of Institute of Public Health, Bangalore (Study ID IEC-FR/03/2018 vide IEC letter number IPH/18-19/E/226 dated 5 ^th^ July 2018 valid till July 2019; renewed vide IEC IPH/19-20/E/183 valid till March 2020) . Relevant portions of phase 1 that relate to biomedical data collection have also received ethics clearance from Mysore Medical College and Research Institute (vide letter from ethics committee dated 2 August 2018). Ethics approval procedures are ongoing for phases 2 and 3. Extended data related to ancillary care, problems foreseen in the conduct of the study, data management and quality are available (see *Extended data*) ^40^. The ancillary care plan outlines course of action to be undertaken when particular health problems are either reported to or witnessed incidentally in/around households visited by the study team data collectors during the household survey or any other visit related to data collection in course of phases 2 and 3. Given that the project study sites are in remote locations with limited earlier efforts and experiences with conducting surveys, potential problems foreseen in the conduct of the study have been outlined. These pertain to the logistics of conducting study in sites that are very far from each other, preparations needed to obtain relevant permissions to enter protected forest areas and measures to be taken in case of high refusal rates at the sites.

## Dissemination

The study involves close consultations, discussions and participation by several local (district and community-based) and state level actors in the southern Indian study site. Broadly, the dissemination will focus on public engagement and policy engagement.

Under our policy engagement strategy, we will organize multiple district-level meetings every year with implementers and community-based organisation representatives to share findings as and when they are available. This will include meetings to discuss survey findings (objective 1), to discuss the case study findings and challenge the middle range theories (objective 2), and finally co-create an intervention. State level engagement will include the preparation of a policy engagement plan that will begin with the formulation of a specific objective (based on the results) and strategies to achieve this policy change objective. We shall seek policymaker and implementer involvement early on in the project to avoid approaching them as passive consumers of research data, and rather invite them as active participants in the research (especially in objectives 2 and 3). We will work closely with tribal affairs, health, forests and women-child development departments at the state level.

Public engagement shall focus on making anonymised data and appropriate visualisations publicly available through open-data initiatives and platforms. We will also explore the possibility of involving local tribal youth in photo stories and facilitate opportunities for local folk art to engage with research findings related to health inequities through attempting to facilitate research themes to be integrated into local theatre/art forms.

## Study status

The study phase 1 has now completed data collection in three sites and data collection is ongoing in two more sites; phase 1 data collection is expected to be completed by December 2020. Ethics clearance for phases 2 and 3 are in final stages and data collection for phase 2 is expected to begin January 2020. Phase 3 is expected to begin in June 2020. 

## Discussion

Current strategies for improving tribal health draw upon the experience of vibrant (but geographically limited) NGOs and civil society. There are inspiring examples of organisations that have done pioneering work both in service delivery as well as activism/advocacy in geographically remote areas and among socially vulnerable communities. While these are valuable and could inspire new thinking about the nature of engagement with communities, systematic and participatory research embedded within forested areas with tribal population is still limited to few locations and organisations. The THETA study aims to initiate a multi-institutional and multi-stakeholder tribal health research and action agenda in southern Karnataka.

Partly, poor health outcomes among Indian tribal communities can be attributed to poor availability and quality of information on access and utilisation of health services, illness profiles, and health-seeking behaviour ^6^. However, the availability of information to plan and manage health services for designing contextually relevant public health interventions is lacking. Whether their poor health status is due to their remote location or if, and how, social disadvantage plays a role in this is less well understood. Wherever systematic and historical social disadvantages exist, they in turn create adverse societal conditions that prevent these populations or sub-groups from realising individual measures to overcome health or social inequalities. In this sense, the existence of any social disadvantage is an essential pre-requisite for inequity. Hence, it is important to understand the role of social disadvantage in driving poorer access, utilisation, and health outcomes among tribal communities in order to achieve equitable health.

In the current proposal, we seek to build upon these preliminary insights from the field from the literature on tribal health in India. Among the determinants of tribal health, environmental and social determinants are less well studied ^14^. Further, there is limited research on “the pathways through which health inequities are created, and the political or policy environment that facilitates the processes” ^41^. Similarly, research on interventions either in health systems or among communities that mitigates health inequities are scarce ^5, 6, 41^. Among the social determinants of tribal health, geographical remoteness, proximity to forest areas, cultural distance from the “mainstream” population, historical isolation and social stratification have all been postulated to have a significant effect on their health outcomes. However, a global explanation, lumping together all these social determinants will not address the specific differences within and across tribal and non-tribal communities. Understanding the specific nature of these interactions within particular contexts helps implementers and planners in improving access and utilisation to health services and planning equitable interventions in tribal populations. Especially in tribal health, the social determinants related to land ownership and access to forest resources, roads, and other amenities also have implications for forest conservation and are expected to be outcomes of a negotiated dynamic between restrictive forest protection legislation on the one hand and enabling tribal development policies and initiatives on the other ^2, 9^. We seek to explore how tribal health is a negotiated outcome as a result of localised interaction between geographical and social factors. This includes examining local power dynamics within and across tribal and non-tribal communities and socio-political factors.

## Data availability

### Underlying data

No underlying data are associated with this article.

### Extended data

Figshare: Comparison of demographic, health and nutrition indicators between scheduled tribe (ST) and non-ST population across six states in India complied from various sources. https://doi.org/10.6084/m9.figshare.10028804.v3 ^7^

Figshare: THETA project: Ancillary care, problems foreseen and quality. https://doi.org/10.6084/m9.figshare.10292999.v1 ^40^

Figshare: THETA tribal health survey questionnaire including list of modules in the survey tool, their sources and intended respondents. https://doi.org/10.6084/m9.figshare.10292963.v1 ^23^

Data are available under the terms of the Creative Commons Attribution 4.0 International license (CC-BY 4.0).
