# Supplementary material for: Towards Health Equity and Transformative Action on tribal health (THETA) study to describe, explain and act on tribal health inequities in India: A health systems research study protocol
Source: Wellcome Open Res. 2019 Dec 13;4:202. [Version 1] doi: 10.12688/wellcomeopenres.15549.1 (PMC7076281; doi:10.12688/wellcomeopenres.15549.1)
Supplement: Peer review history

# Peer review: Reviewer response for version 1

**DOI:** 10.21956/wellcomeopenres.17026.r38001

**Published:** 2020-03-11

**Authors:** Prathibha Ganesan

**Affiliations:** 1 Tata Institute of Social Sciences, Mumbai, Maharashtra, India

The proposed study on health equity and transformative action among forest-dwelling tribal communities ​is​ relevant and significant in the Indian context​. The study is meticulously planned and clearly articulated.

 ​Few suggestions: 

1. I find a lack of clarity in the selection of tribal communities for the study. Here, researchers are studying the forest associated tribal communities ( *Adivasis*), who are living in remote areas. The forest associated Adivasi tribal communities may be mostly dependent on forest produce, and socially and culturally different from (a) the other tribal communities (who live closer to the non-tribal settlements), and b) the non-tribal communities. For example, the *Kattu paniya* community who is a forest-dwelling and forest-dependent tribal community are very different from *the Nattu paniya* community who work in the fields as agricultural laborers in Kerala.​ The research paper does not give a clear idea about this kind of differentiation within the tribal communities. Also, in table one, the researchers have given names of tribes in the study area. However, in my knowledge, not all the tribes in the table (at least from Kerala) are not forest associated communities. For example, *Adiya* tribes are not forest dependent or forest associated but agricultural laborers. Therefore, it will be good if the researchers can define forest-dwelling/forest associated communities and provide a rationale for choosing each tribal community that is given in the table.
2. The socio-cultural aspects of tribal communities are the most significant determinants of their health-seeking behavior. It is a common knowledge that modern health care seeking behavior of tribal communities is weaker when compared to non-tribal communities. In such a situation, understanding and comparing the health status of tribal and non-tribal communities may not be very helpful. However, understanding the healthcare-seeking behavior and access issues of the two types of tribal communities (forest-dwelling ​(Adivasis)​ and non-forest dependent​ tribals​) may give much more insight into the socio-cultural dynamics of the communities. The researchers may consider this aspect while considering research objective one or purposively choose cases to do in-depth research in the second objective.

I confirm that I have read this submission and believe that I have an appropriate level of expertise to confirm that it is of an acceptable scientific standard.

# Peer review: Reviewer response for version 1

**DOI:** 10.21956/wellcomeopenres.17026.r37446

**Published:** 2020-01-20

**Authors:** Shridhar Kadam

**Affiliations:** 1 Indian Institute of Public Health, Bhubaneswar, Odisha, India

The proposed study is very important, relevant and crucial to explore explanations of inequities in health care access and health outcomes among the people living in hard-to-reach areas and tribal communities as compared with other population sub-groups.

 The researchers will need to assess and reconsider the feasibility of data collection, especially undertaking the lab investigations in the context of conducting research in hard-to-reach areas and in a tribal population.

 I am not an expert of calculation of sample size, however, the researchers should take comments and inputs from relevant experts.

I confirm that I have read this submission and believe that I have an appropriate level of expertise to confirm that it is of an acceptable scientific standard.
